# “Cured” patients with early-onset idiopathic scoliosis after serial casting are at risk of recurrence at intermediate follow-up

**DOI:** 10.1007/s43390-025-01092-1

**Published:** 2025-05-03

**Authors:** Rayyan Abid, Abigail E. Manning, Peter F. Sturm, Ying Li, Craig M. Birch, Michal Szczodry, Michael P. Glotzbecker

**Affiliations:** 1 Department of Orthopaedic Surgery, Rainbow Babies & Children’s, Case Western Reserve University/University Hospitals, 2101 Adelbert Rd, Cleveland, OH 44106 USA; 2 University of Washington School of Medicine, Seattle, WA USA; 3 Cincinnati Children’s Hospital, Cincinnati, OH USA; 4 C.S. Mott Children’s Hospital, Michigan Medicine, Ann Arbor, MI USA; 5 Boston Children’s Hospital, Boston, MA USA; 6 Shriners Hospitals for Children-Chicago, Chicago, IL USA

**Keywords:** Early-onset scoliosis, Casting, Bracing, Non-operative techniques

## Abstract

**Purpose:**

Serial casting limits curve progression while preserving spinal growth, delaying or even eliminating the need for surgery. Some patients with EOIS can be “cured” with curve reduction under 15°. However, no long-term studies have defined whether “cured” patients maintain small curves or if they are at risk of progression. We examined if casting patients remained “cured” following treatment.

**Methods:**

We identified 40 EOIS patients who were treated with serial casting, achieved curves under 15° and had minimum 2 years of follow-up after completing the treatment. Failure was defined as an increase > 6° resulting in a curve magnitude > 15° at any point during follow-up, requiring cast/brace treatment after cessation of initial cast/brace, or undergoing surgery. Average curve magnitude at the time of cure was 11.1°. Kaplan–Meier survival analysis was used to identify failure rates over time.

**Results:**

10 patients (25.0%) met criteria for failure. Mean time from cure to last follow-up was 4.3 years. 3 patients (7.5%) completed bracing and were later re-braced while 2 (5.0%) required surgery. Mean curve magnitude of “failed” patients was 27.4° with an average increase of 15.6°. At 5.1 years, probability of successful treatment is 64.2%. For “failed” patients, median time to failure was 2.4 years. Successful patients were braced for median 1.4 years, while “failed” patients had a median of 1 year.

**Conclusion:**

While EOIS patients may be “cured” with serial casting, this may not be sustained. The percentage of “failures” likely will increase with longer follow-up through skeletal maturity, and patients must be closely monitored after concluding casting/bracing.

## Introduction

Early-onset idiopathic scoliosis (EOIS) refers to scoliosis affecting children less than 10 years of age [1]. EOIS can result in a number of consequences including severe cardiopulmonary development issues, abnormal growth of the trunk, and thoracic insufficiency syndrome [2, 3]. Before the advent of new procedures, spinal fusion was the most common treatment for EOIS, but its association with thoracic insufficiency syndrome led to the development of newer techniques [4]. However, these newer surgical treatments are also frequently associated with complications [5–9]. Earlier treatment has also been shown to be associated with more complications than when treatment can be delayed. For example, in a study of 140 patients, Mehta found that 58% experienced at least one complication, and growing rod procedures exhibit comparable complication rates [6]. Similarly, it appears that these lengthening procedures become decreasingly effective over time and there is a law of diminishing returns, suggesting that it is preferable to delay these procedures as long as possible [10].

Serial casting has long been described as an effective growth-delaying technique [4]. Existing studies have shown that serial casting can be a viable alternative to surgical techniques [11]. Moreover, these studies still typically classify casting as a precursor to more invasive treatment rather than a cure in its own right. More specifically, if a child exhibits a small curve (> 25° and progression > 10°), then casting is often prescribed before performing surgery [4, 11]. In some children, serial casting can be used to cure scoliosis. Mehta has demonstrated that serial casting can serve as a viable cure, but it is dependent on the age at the onset of casting [12]. Similarly, Sanders et al. had success casting children under 20 months, bringing 55 patients with Major curve magnitudes greater than 60° to full correction [13].

Regardless, there are relatively few studies that offer long-term follow-ups of patients following the conclusion of serial casting and consider potential risk factors for curve recurrence after the end of treatment. It is important to understand the chance of recurrence rate to counsel parents but also to determine the need for follow-up screening protocols. The purpose of our study was to confirm whether patients treated by serial casting to “cure” maintained curve correction over time. For those that recurred, we aimed to study potential risk factors for curve progression after completion of treatment.

## Methods

**Fig. 1:**
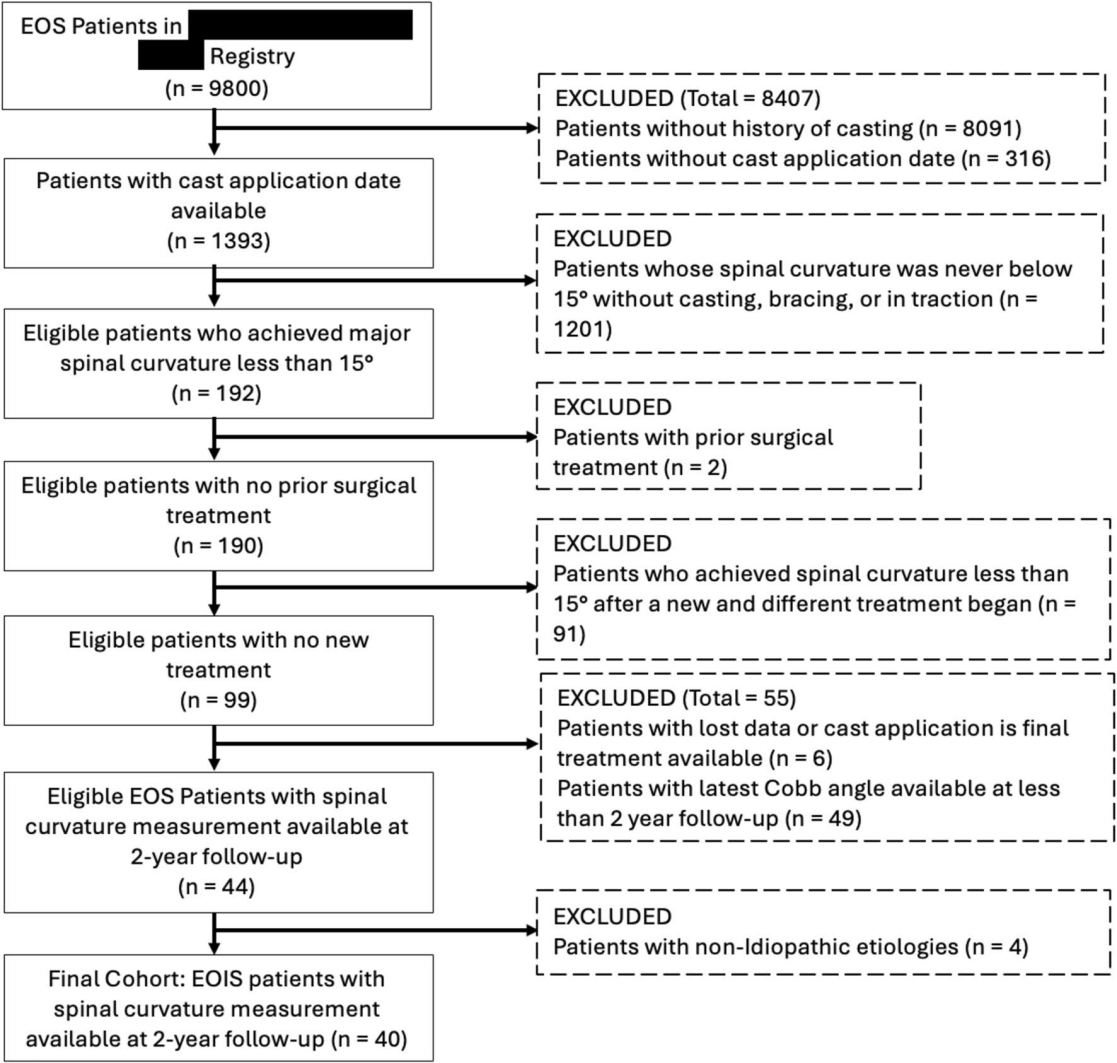
STROBE diagram depicting patient selection process

A query of a multicenter database identified 40 patients with early-onset idiopathic scoliosis who were treated with serial casting, achieved a curve under 15°, and had a minimum of 2 years of follow-up after reaching this reduced curvature. Exclusion criteria (Fig. 1) included the presence of non-idiopathic etiologies (syndromic, congenital, or neuromuscular). The average age of these patients at the time of first cast application was 1.3 years (0.37–3.58).

The collected demographics were gender, race, comorbidities, age at application of first cast, total number of casts applied, age at termination of casting, nature of subsequent treatment, duration of bracing after removal of the cast, Major curve magnitude at cure, Major curve magnitude at latest follow-up, date of most recent X-ray, pre-cast Major curve magnitude, and pre-cast sagittal kyphosis.

Our criteria for recurrence included an increase in Major curve magnitude of > 6° resulting in a Major curve magnitude of > 15° at any point during the study and/or requiring a cast/brace or surgical treatment after the cessation of the initial cast/brace [14–16].

To identify recurrence rates over the duration of the study, a Kaplan–Meier survival analysis was applied to the sample. Since this was a retrospective study and we were limited by the number of patients who met our criteria, no power analysis was conducted. Sagittal kyphosis was excluded from our analysis due to insufficient measurements. All statistical analyses and figures were done using R software (version 4.4.1 for macOS).

## Results

The average number of casts applied to the 40 patients meeting inclusion criteria was 5.6 (2–17 casts), and the mean time from the first cast application to the date of “cured” curvature was 1.2 years (0.15–3.78 years). The mean time between the date of cure and latest follow-up was 4.3 years (2.1–11.0 years). The mean Major curve magnitude before treatment was 34.0 degrees (6°–100°) and the mean Major curve magnitude at the time of “cure” was 11.1 degrees (1°–15°). The mean Major curve magnitude at latest follow-up was 14.3° (0°–71°).

**Fig. 2:**
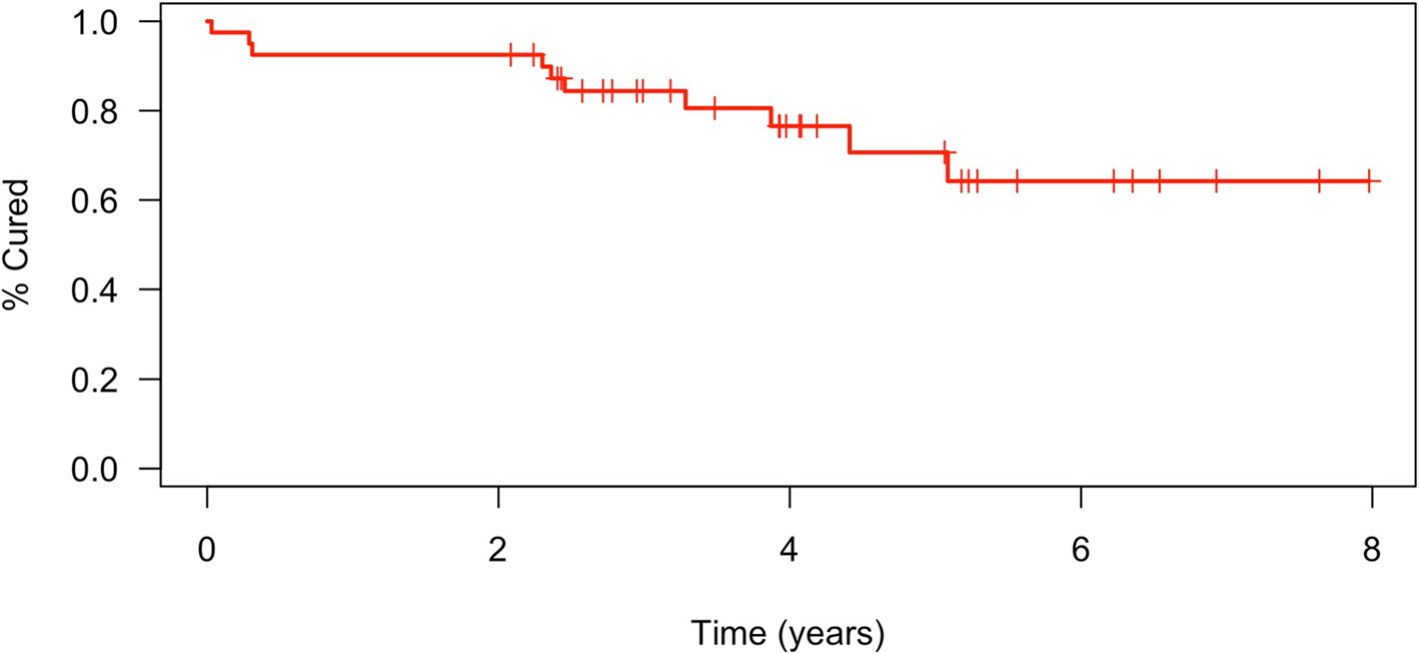
Kaplan–Meier survival curve demonstrating the percentage of patients that had curve progression or required a secondary treatment over time after being successfully treated in a cast for EOIS

Of the 40 patients, 37 (92.5%) received bracing following termination of casting. 10 (25.0%) met our criteria for recurrence, with 3 patients (7.5%) being re-braced following completion of initial bracing and 2 (5.0%) requiring surgery with growth-friendly instrumentation. One surgical patient was not braced while the other was braced only once before receiving VEPTR or traditional growing rods. The remaining 5 (12.5%) patients met our radiographic definition of recurrence but did not require further treatment as of their latest available follow-up. In the 10 patients who met our criteria for recurrence, the mean curve magnitude at latest follow-up was 27.4 degrees (8°–71°) with an average curve progression of 15.6° (− 7° to 60°). The median time to recurrence in our sample was 2.4 years. Our Kaplan–Meier Analysis (Fig. 2), predicts that, at 5.1 years of follow-up, the probability of successful treatment is 64.2% (95% CI 47.1, 87.6).

14 patients (35.0%) were braced for > 2 years following casting. Patients with successful treatment were braced for a median of 1.4 years while patients who had recurrence after treatment had a median brace time of 1 year. 2 cured patients (5.0%) received no bracing, achieving a sufficiently reduced curvature with casting alone. 1 failed patient (2.5%) did not undergo bracing and instead received surgical treatment.

Generally, there appeared to be little variation in rates of recurrence or time to recurrence based on a variety of risk factors (Table 1). 5 of the failed patients were female and 5 were male. Females had a mean time to recurrence of 3.1 years, while males had 3.2 years. Of the cured patients without recurrence, 15 were female and 15 were male, and cured patients had an average follow-up length of 6.0 years. Before undergoing treatment, patients who met our criteria for recurrence had a mean Major curve magnitude of 38.8°, while cured patients had an angle of 32.4°, and this difference was not significant. Cured patients took an average of 1.3 years to reduce their curves to 15° while failed patients took 1.1 years. Cured patients spent an average of 1.0 years in casts and patients with recurrence spent an average of 1.4. Cured patients began casting at 1.2 years, while failed patients began casting at 1.5. None of these differences were significant (*p* < 0.05), and these comparisons can be found in Table 1.

**Table 1:** Unpaired t-tests demonstrating that the two-tailed P values were not statistically significant for any of the examined risk factors

Risk factor	Cured	Failed	p value
Sex			
Male	15	5	N/a
Female	15	5	N/a
Mean major curve magnitude at treatment initiation (degrees)	32.4	38.8	0.492
Mean time in cast (year)	1.0	1.4	0.298
Mean age at treatment initiation (years)	1.2	1.5	0.363

## Discussion

There exists great diversity in the methodologies of treatment for EOIS with the most invasive methods carrying the greatest risk [17, 18]. Anterior and posterior spinal fusions were once thought to be the most viable form of treatment, but these procedures carry significant complications for patients including reduced lung capacity and spinal growth, significantly increasing mortality [18, 19]. More recently developed surgeries can preserve lung growth while controlling curve progression [20, 21]. However, we have learned that these procedures still carry a high risk of complications, and the risk of complications may increase if surgical treatment is initiated earlier [6, 7, 22]. Moreover, prolonged management of surgical treatments for EOIS is associated with greater amounts of complications because each lengthening introduces new opportunities for complications to arise.

A lower risk, less invasive form of treatment for EOIS such as casting, can delay surgical treatment and in some cases “cure” the scoliosis with reduction of the curve under 15°. For example, Sanders et al. was able to reverse the curve of 49 EOS patients in a sample of 55 children (89.1%) and followed up with patients for a year after the initiation of casting. For patients with curves less than 60° who began casting at an average of 1.1 years, casting typically resulted in full correction [13]. Fletcher et al. performed a retrospective study with 29 patients, reporting on an older population, initiating casting an average age of 4.4 years. This sample had an average Major curve magnitude of 68.8° and found that, after a mean follow-up time of 5.5 years from initial casting, surgery could be delayed by an average of 39 months in about half of patients, but a cure should not be expected [11]. However, the viability of serial casting as a cure for EOIS has also been shown.

Most notably, Mehta performed a prospective study of 136 EOIS patients under the age of 4 years with infantile scoliosis [12]. In Group 1, 94 children treated in the early stages of progression, the scoliosis was resolved at a mean age of 3 years and 6 months. These patients began treatment at a mean age of 1 year and 7 months, began with a mean Major curve magnitude of 32°, and none required surgery after following up at an average age of 11 years and 2 months. Interestingly, only 2 of these patients were documented to have relapsed, exhibiting greater treatment success over a longer period of time. However, Mehta could only reduce but not reverse deformity in Group 2 which included the remaining 42 patients who were referred at a greater age (2 years and 6 months) [12].

While the failure to reverse curves in Group 2 of Mehta’s study makes it difficult to draw comparisons to our own cohort, Group 1 offers a more similar sample. In both cohorts, children began casting at approximately 1.5 years, and follow-up lasted multiple years. Interestingly, our results suggest the need for more cautionary management and monitoring of these curves as patients approach skeletal maturity. After only 5 years, we expect that over a quarter of patients’ curves should expect their treatment to “fail”, either experiencing curve regression or further unintended treatments, whereas Mehta’s population had only 2 of 94 patients (2.1%) recur after more than a decade [12].

More recent studies, however, appear to align more closely with our own design and results. Fedorak et al., for example, conducted follow-ups on 38 patients with EOIS for a minimum of 5 years [16]. These follow-ups were conducted at the initiation of casting rather than the termination of casting, but were, on average, longer than our own at approximately 8 years. While this follow-up length was not long enough to analyze how curves or treatment plans changed as patients approached skeletal maturity, they did identify 3 patients (7.9%) whose curves relapsed above 15°. However, Fedorak et al. also predict that, as these patients approach skeletal maturity, rates of success will decline and the number of those requiring surgery or further treatment will increase [16].

Regan et al. also offer a study with results similar to our own. In a sample of 21 patients with an average 7-year follow-up beginning at the initiation of casting, 13 patients met their criteria for successful correction [23]. However, 3 of these patients (23.1%) experienced significant regression as they began puberty. Each of these patients continued casting through 7 years of age, but a lack of adherence to bracing protocol caused their curves to regress past their original deformity [23]. This timeframe and rate of regression roughly agrees with our own as we hypothesized that nearly 40% of patients would fail 5 years after the termination of casting and bracing, approximately the time when puberty would begin.

Although we concluded that casting may “cure” patients with EOIS, it may indeed not be a sustained treatment for all patients over time. Within our sample of patients “cured” at the beginning of their follow-ups, 25% met our criteria of recurrence, and 12.5% received some form of additional treatment. Even after achieving a small curve, many patients continued to brace for an extended period of time. Thus, patients with EOIS and their families should expect that further treatments and bracing may be required even after their Major curve magnitude is reduced below 15° with serial casting. As this study did not follow all patients to skeletal maturity, as time passes from the conclusion of bracing and patients begin to reach skeletal maturity, we expect that the percentage of patients experiencing recurrence will likely increase. Thus, regular follow-ups and close monitoring will be necessary after the completion of casting and bracing.

Similar to other studies on this topic, the limitations of our study include the small sample size and its retrospective design. Longer follow-up times, and follow-up to skeletal maturity, would also provide more insight into the long-term viability of serial casting following the termination of bracing. Utilization of a multicenter database also presents a number of drawbacks, including variation in treatment protocols among participating institutions and potential bias in the selection of patients in our sample. Moreover, comparisons between failed and “cured” patients may have been obscured by a lack of data regarding other risk factors such as apical vertebral rotation and pelvic obliquity. Variability in duration of bracing could have also influenced this relationship, and difficulty monitoring brace compliance likely has a similar effect.

Regardless, this study offers surgeons, patients and their families a look at what the expected effectiveness and timeframe for serial casting as a treatment for EOIS may be. While patients can be “cured” by serial casting, curve progression may occur as patients continue to grow. 25% of our sample experienced failure of their treatment at an average of 2.4 years with some requiring further bracing and others opting for surgery. We could not identify any risk factors that might help us predict who might recur. Therefore, parents should be counseled that children may require further treatment even after they are successfully cured with serial casting. These patients also require ongoing, long-term screening and follow-up to detect recurrence.

## Data Availability

The data that support these findings are available from the corresponding author upon reasonable request.
